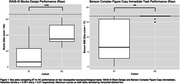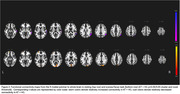# Functional Connectivity of the Affective Blindsight Pathway in Atypical Alzheimer’s Disease

**DOI:** 10.1002/alz.090503

**Published:** 2025-01-03

**Authors:** Hae Young H Yi, Joyce S Li, Anne E Trainer, Bronte Ficek‐Tani, Todd Constable, Carolyn Fredericks

**Affiliations:** ^1^ Yale School of Medicine, New Haven, CT USA

## Abstract

**Background:**

Affective cognition and emotion processing is impaired in amnestic Alzheimer’s disease (AD), although less is known about atypical (AT) variants such as logopenic variant primary progressive aphasia (lvPPA) and posterior cortical atrophy (PCA). The affective blindsight pathway bypasses V1 via the superior colliculus‐pulvinar route to activate the amygdala in cases of occipital lesioning and may explain maintenance of emotion identification and visual information processing in non‐amnestic AD despite atrophy in visuospatial regions. We sought to characterize functional connectivity from key regions along the affective blindsight pathway in a clinically heterogeneous AD cohort.

**Method:**

We collected structural, resting‐state, and task functional MRI scans where participants viewed affective face and neutral scene images, and neuropsychological data in individuals with clinically heterogeneous AD (8 lvPPA, 9 PCA) and age‐matched healthy controls (24 HC). We assessed connectivity from 4mm radius seeds located along the affective blindsight pathway (superior colliculus, medial pulvinar, and amygdala) using CONN toolbox. Two‐sample t‐tests were used to compute group‐level differences (AT > HC; p < 0.05/0.05), using w‐scores to correct for atrophy.

**Result:**

As expected, AT participants performed significantly worse on visuospatial tasks outside the scanner (WAIS‐III Blocks Design, Benson Complex Figure Copy immediate recall; p < 0.01). During resting state scans, the AT group had relatively lower connectivity to visuospatial regions including the lateral occipital cortex, angular gyrus, and precuneus from the superior colliculus and medial pulvinar seeds. During the scenes/faces task, they displayed relatively higher connectivity to those same visuospatial regions. From the amygdala seed, the AT group had higher connectivity to area V1 during the resting state scan and lower connectivity to frontal lobe regions during scenes/faces.

**Conclusion:**

We explored functional connectivity of the affective blindsight pathway in a clinically heterogeneous AD cohort. Connectivity was relatively higher in the AT group from the superior colliculus, medial pulvinar, and amygdala to occipital lobe regions commonly atrophied in PCA and lvPPA, suggesting a function of this pathway may be to compensate for disease‐related atrophy. While preliminary, these findings hint that the blindsight pathway may serve a compensatory function in atypical AD.